# Boosting of tau protein aggregation by *CD40* and *CD48* gene expression in Alzheimer's disease

**DOI:** 10.1096/fj.202201197R

**Published:** 2022-12-15

**Authors:** Sung‐Hyun Kim, Key‐Hwan Lim, Sumin Yang, Jae‐Yeol Joo

**Affiliations:** ^1^ Department of Pharmacy College of Pharmacy, Hanyang University Ansan Republic of Korea; ^2^ Neurodegenerative Disease Research Group Korea Brain Research Institute Daegu Republic of Korea; ^3^ Department of Pharmacy College of Pharmacy, Chungbuk National University Cheongju‐si Republic of Korea

**Keywords:** Alzheimer's disease, biomarker, *CD40*, *CD48*, GWAS, tau

## Abstract

Neurodegenerative diseases result from the interplay of abnormal gene expression and various pathological factors. Therefore, a disease‐specific integrative genetic approach is required to understand the complexities and causes of target diseases. Recent studies have identified the correlation between genes encoding several transmembrane proteins, such as the cluster of differentiation (CD) and Alzheimer's disease (AD) pathogenesis. In this study, *CD48* and *CD40* gene expression in AD, a neurodegenerative disease, was analyzed to infer this link. Total RNA sequencing was performed using an Alzheimer's disease mouse model brain and blood, and gene expression was determined using a genome‐wide association study (GWAS). We observed a marked elevation of *CD48* and *CD40* genes in Alzheimer's disease. Indeed, the upregulation of both *CD48* and *CD40* genes was significantly increased in the severe Alzheimer's disease group. With the elevation of *CD48* and *CD40* genes in Alzheimer's disease, associations of protein levels were also markedly increased in tissues. In addition, overexpression of *CD48* and *CD40* genes triggered tau aggregation, and co‐expression of these genes accelerated aggregation. The nuclear factor kappa B (NF‐ĸB) signaling pathway was enriched by *CD48* and *CD40* gene expression: it was also associated with tau pathology. Our data suggested that the *CD48* and *CD40* genes are novel AD‐related genes, and this approach may be useful as a diagnostic or therapeutic target for the disease.

**Abbreviations**
5xFAD: 5 familial AD mutations
AD: Alzheimer's disease
ApoE: apolipoprotein E
Aβ: amyloid β
CD: cluster of differentiation
CFP: cyan fluorescent protein
DEGs: differentially expressed genes
FPKM: fragments per kilobase per million reads
FRET: fluorescence resonance energy transfer
GEO: gene expression omnibus
GFP: green fluorescent protein
GWAS: genome‐wide association study
IĸBα: inhibitor of kappa B alpha
NF‐ĸB: nuclear factor kappa B
PD: Parkinson's disease
PPI: protein–protein interaction
RT‐PCR: reverse transcription polymerase chain reaction
UCSC: University of California Santa Cruz
WT: wild‐type
YFP: yellow fluorescent protein

## 1 INTRODUCTION

In the past few years, disease‐related risk genes and mutations have been revealed through the rapid and surprising development of next‐generation sequencing technology from a large database, and genome‐based studies have been able to unveil the genetic factors for targeted diseases.^1^, ^2^ Genome‐wide association study (GWAS) greatly improved the genetic characteristics of abnormal variations and/or transcriptions in diseases.^3^, ^4^ GWAS conducted using large genetic databases reveal accurate information about target genes that cause various human disorders, and thus it has been applied in the discovery of disease‐related factors that are involved in important functions and progression of diseases such as cancer, Alzheimer's disease (AD), and Parkinson's disease (PD).^5^, ^6^, ^7^, ^8^ GWAS have already been used to analyze abnormally expressed genes that have the potential to serve as biomarkers for the diagnosis and/or treatment of AD. Thus, recent GWAS have greatly increased the identification of AD‐associated genes.^9^, ^10^, ^11^

AD is the most common type of neurodegenerative disease, accounting for approximately 60%–70% of dementia cases and characterized by symptoms such as cognitive dysfunction, loss of functional abilities, behavioral changes, and loss of memory.^12^ In 2021, over 55 million people worldwide had dementia, and the number is expected to increase to 78 million by 2030.^13^, ^14^ Therefore, many studies have investigated the crucial AD‐related risk factors to demonstrate the mechanism of AD progression and develop an accurate diagnosis of AD patients. Many studies have identified initially in AD risk factors research and demonstrate the AD‐specific mutation genes, such as the tau, apolipoprotein E (ApoE), and amyloid β (Aβ).^15^ It is likely that ApoE and Aβ are indispensable for AD progression; tau also has been regarded as a strongly associated protein.^16^, ^17^ Tau is a microtubule‐associated protein involved in intracellular transport, development of cell processes, and establishment of cell polarity through stabilized neuronal microtubules. However, tau hyperphosphorylation promotes abnormal aggregation as neurofibrillary tangles, causing impairment of neuronal cells in AD. Therefore, aggregation of tau has been identified as one of the major hallmarks of AD.^18^, ^19^

Cluster of differentiation (CD) proteins, a family of cell surface molecules, acts as a factor of identification for immunophenotyping of cells by providing targets. CDs have revealed up to 371 families in humans and were assigned a different number based on the characterizations and functions of the cell. CDs can act as receptors and ligands to initiate a cascade of signaling pathways; thus, the behavior of the cells changes during physiology. Several CDs do not have a role in cell signaling; instead, they are involved in other processes such as cell adhesion. In addition, CDs are associated with humoral and cellular immune responses.^20^, ^21^ In the last decade, several studies have demonstrated that CDs exist in various cell types, and dysregulated expression of CDs contributes to numerous human diseases, including cancer, AD, and PD.^22^, ^23^, ^24^, ^25^, ^26^, ^27^ Therefore, CDs have the potential to serve as diagnostic and therapeutic factors in various human diseases. However, there are not enough studies on the correlation between the expression of AD‐specific CDs and tau.

In this study, we focused on the findings of novel AD‐specific genes and identified upregulation of the *CD48* and *CD40* genes in the tissue and blood of AD mouse models as well as the hippocampus of AD patients through in silico analysis and GWAS. We also demonstrated that *CD48* and *CD40* genes boosted tau aggregation and activated the nuclear factor kappa B (NF‐ĸB) signaling pathways. In addition, we developed a liquid biopsy method, the multiplex reverse transcription‐polymerase chain reaction (RT‐PCR), for rapid and accurate diagnosis in the blood of AD models. Therefore, our findings identified potent novel AD‐related risk factors, *CD48* and *CD40* genes, which may serve as new clinical diagnostic targets for the disease.

## 2 MATERIALS AND METHODS

### 2.1 Cell culture and transfection

Tau RD P301S FRET biosensor cells (CRL‐3275™) were obtained from the American Type Culture Collection (ATCC). Cells were cultured in Dulbecco's Modified Eagle's Medium (DMEM) (Gibco) supplemented with 10% FBS, and 1% penicillin/streptomycin (Hyclone) in 5% CO_2_, 37°C. Cell transfection was performed with gene expression vectors that encode *CD48* and *CD40* with V5 epitope‐tag using Lipofectamine™ 2000 reagent (Invitrogen) and Opti‐MEM (Gibco). Human brain whole tissue lysate of AD patient was purchased from Novus biologicals (Novus).

### 2.2 Animals

5хFAD (4, 6 and 10‐month) transgenic mice, used as models for Alzheimer's disease, were obtained from the Jackson Laboratory. All animal experiments performed in this study were reviewed and approved by the IACUC committee at the Korea Brain Research Institute (KBRI, IACUC‐20‐00018).

### 2.3 RNA sequencing (RNA‐seq)

RNA sequencing data analysis was performed as previously described.^10^, ^11^, ^28^ Briefly, total RNA isolation from mouse cortex and blood (6‐month) was conducted according to the commercial TRIzol reagent protocol (Invitrogen). RNA‐seq libraries preparation was performed based on the Illumina platform, TruSeq Standed Total RNA LT Sample Prep Kit (Human Mouse Rat). Purified RNA was first fragmented and then synthesized into first and second strands of cDNA. The constructed libraries were qualified using Agilent 2100 Bioanalyzer and sequenced using HiSeq™ 4000.

### 2.4 Data filtering and DEG analysis

Base calling derived nucleotide sequences (FASTQ format files) were filtered to separate dirty reads from raw reads. The reads went through standard quality control (QC) criteria. Then, filtered clean reads stored as FASTQ format had aligned on annotated reference genome. The matching reads were counted with HTSeq‐count; for quantifying each gene expression, assembled transcripts were estimated by fragments per kilobase per million reads (FPKM) values using Cufflinks package (v2.2.1). The differentially expressed genes’ (DEGs) analysis including read count filter and fold change analysis was performed using DESeq2 (v1.22.2) and R packages (v3.6.1). Heatmap with hierarchical clustering for gene expression values visualization was performed using DESeq2 and ggplot2 package. The original raw data files are available through the Gene Expression Omnibus (GEO) data set for GSE 151270 and GSE 147792.^11^

### 2.5 Complementary DNA (cDNA) synthesis

Isolated total RNA as templates were used to synthesis complementary DNA (cDNA) according to the manufacturer's protocol. The synthesis for the mature mRNA was performed with PrimeScript™ 1st strand cDNA Synthesis Kit (TaKaRa). To synthesis cDNA for mature mRNA, 1 ug template RNA was used for each with the reaction mixture comprised of PrimeScript RTase, 5х PrimeScript buffer, RNase inhibitor, dNTP mixture, Oligo dT Primer, and nuclease free water. Then, the mixture was incubated under the following conditions: 42°C for 60 min and 95°C for 5 min.

### 2.6 Reverse transcription‐quantitative PCR (RT‐qPCR)

RT‐qPCR was performed following the manufacturer's protocol of SYBR Green PCR Master Mix (Applied Biosystems). Primers employed were *CD48* forward, 5’ GTACAAAGACAATCTTCGAGTC 3′, reverse, 5′ GCACTCTCATGTAGTAGGTAC 3′; *CD40* forward, 5′ AAGTCCCGGATGCGAGCC 3′, reverse, 5′ ATATAGAGAAACACCCCGAAAA 3′; *GAPDH*, forward, 5′ AGGTCGGTGTGAACGGATTT 3′, reverse, 5′ TGTAGACCATGTAGTTGAGG 3′. Each reaction comprises with template cDNA, SYBR Green Master Mix, and forward and reverse primer set: those were adjusted with nuclease free water, then quantified by LightCycler 480 (Roche). Statistical significance was evaluated using an unpaired two‐tailed *t*‐test.

### 2.7 Multiplex RT‐PCR

Multiplex and reverse transcription polymerase chain reaction (RT‐PCR) was performed using Phusion® High‐Fidelity DNA Polymerase (NEB). Each reaction mixture was comprised of components including 5х Phusion HF buffer, 200 μM dNTPs, 1 U Phusion DNA polymerase, template cDNA, nuclease free water, and optimized 0.5 μM two sets of forward/reverse primer mixture (Table 1). The multiplex RT‐PCR amplification condition was optimized for 98°C for 30 s of initial denaturation, followed by 33 cycles of 98°C for 10 s of denaturation, 62°C for 30 s annealing, 72°C for 5 s extension, and then 72°C for 10 min of final extension. The products were visualized on 2% agarose gel.

**TABLE 1:** Primer information for multiplex RT‐PCR

Gene name	Primer sequence (5′ to 3′)	Amplification region	Final concentration (μmol/L)	Size (kb)
*CD48(F)*	CGTATCACCTGGCTTCATAC	Exon 2	0.5	0.211
*CD48(R)*	CCAGGGTTATCTTCAACTCG		0.5	
*CD40(F)*	GCTCTTGAGAAGACCCAATG	Exon 3–5	0.5	0.319
*CD40(R)*	CGAAAAGTGATGACTGATTGG		0.5	

### 2.8 Western blot analysis

Homogenized mouse cortex tissue and cells were lysed in tissue and mammalian protein extraction reagent (M‐PER and T‐PER, Thermo Scientific) supplemented with protease inhibitors cocktail (Cell signaling) on ice. The extracted protein was mixed with 5% of 2‐mercaptoethanol sample buffer and denatured at 100°C. Protein samples were size‐dependently separated by SDS‐PAGE on the 4%–20% gradient gel (Invitrogen) and transferred to a PVDF membrane (Millipore). The membranes were blocked with 5% skim milk in TBS buffer with 0.1% tween and all wash steps were performed using TBS buffer with 0.1% tween. Primary antibodies used for analysis were anti‐CD48 (#sc‐8397, Santa Cruz), anti‐CD40 (#86165, Cell Signaling), anti‐pIκBα (#2859, Cell Signaling), anti‐IκBα (#4812, Cell Signaling), anti‐pNF‐κB (#3033, Cell Signaling), anti‐NF‐κB (#8242, Cell Signaling), anti‐V5 (#13202, Cell Signaling), and anti‐β‐actin (#A300‐491A, BETHYL). Chemiluminescent detection was performed using ECL reaction and ChemiDoc MP imaging system (Bio‐Rad).

### 2.9 Tau seeding and fluorescence resonance energy transfer (FRET) assay

In vitro tau seeding and FRET assay using biosensor cells has been widely characterized and previously described. Briefly, tau RD P301S FRET biosensor are HEK293T cells stably expressed the tau repeat domain of P301S with cyan fluorescent protein (CFP) and yellow fluorescent protein (YFP). It could also detect the tau aggregation as the production of FRET signal.^29^ Cells were cultured at a density of 60–70% in culture media (DMEM with 10% FBS and 1% P/S), and transfected with human protein lysate from the brain of an AD patient or the *CD48* and *CD40* (CD48‐V5 and CD40‐V5) in 24 h. Also, media were changed and incubated for a total of 30 h. Transfection complexes were mixed by adding 3 μg of AD patient brain lysate or *CD48* and *CD40*, and 6 μl of Lipofectamine™ 2000 (Invitrogen) with Opti‐MEM (Gibco) for transfection condition, as previously described.^29^, ^30^ Transfection mixtures were incubated at room temperature for 20 min before being added drop‐wise to each well. After 30 h, Cells were washed with PBS and fixed with 4% paraformaldehyde for 15 min. Cells were mounted by VECTASHIELD HardSet Antifade Mounting Medium with DAPI (Vector Laboratories). Representative images of cells were visualized by confocal microscopy using the 400× oil immersion lens with DAPI and green fluorescent protein (GFP) channel. CFP and FRET signals were measured by SpectraMax‐iD5 microplate reader (Molecular Devices). Also the FRET signal was removed from the background and normalized to the CFP signal.

### 2.10 Microarray and in silico analysis

Human microarray datasets GSE1297 were downloaded from EMBL‐ENA website (www.ebi.ac.uk/ena). The GSE1297 datasets were derived from human hippocampal tissue in Alzheimer's disease patients. Their microarray experiments were performed using Affymetrix Human Genome U133 Plus 2.0 Array (Affymetrix). The obtained raw data from human hippocampus microarray were analyzed by a series of python commands and were visualized with help of the Agilent GeneSpring 7.3. Shortly, raw datasets were normalized by dividing each gene's intensity values by each sample's 50% percentile value. Also, all genes in samples of the same group (normal, incipient, moderate, and severe) were averaged. Also, to calculate the differentially expressed genes (DEGs), the values of average in each group were normalized by the control group. The genes with fold change ratio from 0.5 to 2 were considered as DEGs. Statistical significance was evaluated using post hoc test after one‐way ANOVA. Detailed subject characters are summarized in Table S1.

### 2.11 siRNA sequence and transfection

siRNAs targeting human *CD40* and *CD48* sequences were as follows: *CD40* sense, 5′ GCGAAUUCCUAGACACCUGUU 3′, antisense, 5′ UUCAGGUGUCUAGGAAUUCGC 3′^31^; *CD48* sense, 5′ GGUGACCAGCAUUCAAGGU 3′, antisense, 5′ ACCUUGAAUGCUGGUCACC 3′. Negative siRNA duplex (Bioneer) was used as a control. The indicated cells were used, and each transfection was performed according to the manufacturer's protocol using Lipofectamine™ 2000 (Invitrogen) to be 100 nM of siRNA into cells. Cells were co‐overexpressed with both *CD40* and *CD48* for 24 h, then were harvested after 48 h of siRNA co‐transfection. Single siRNA against *CD40* was used to knockdown the endogenous *CD40* through 48 h of incubation. Knockdown efficiency was confirmed after 48 h of siRNA treatment incubation by immunoblotting.

### 2.12 Statistical analysis

All statistical analyses of experiment were performed using GraphPad Prism version 9 (GraphPad Software) for statistical significance. The data were presented as the mean ± standard error of the mean (SEM). Statistical differences were determined by the one‐way ANOVA or Student *t*‐test (unpaired two‐tailed). Results with a value of *p < .05, **p < .01, and ***p < .001 were considered statistically significant.

## 3 RESULTS

### 3.1 Gene expression analysis of *CD48* and *CD40* based total RNA‐seq from in AD mouse model

**FIGURE 1:**
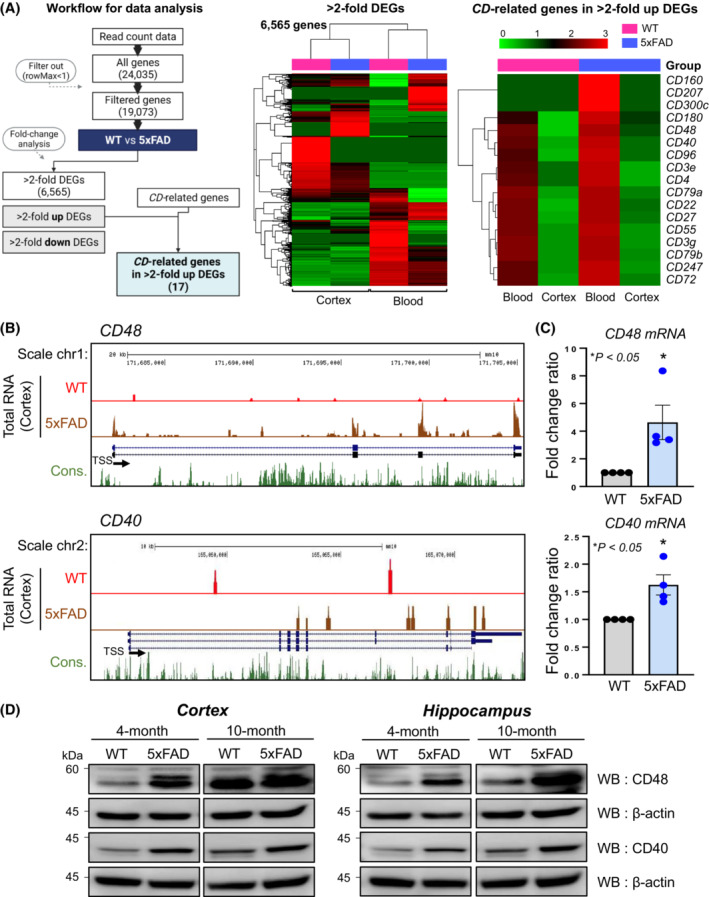
RNA‐seq based GWAS for expression analysis profile of *CD48* and *CD40* in AD mouse model. (A) Gene expression level was identified by presented RNA‐seq data analysis workflow. Heatmap visualizes genes expression value with intensity after log2 scale for the fold change analysis. Each indicates the >2‐fold differentially expressed genes (DEGs) within WT and 5xFAD sample comparison, and among that of *CD*‐related genes. The samples on the columns were derived from cortex and blood. (B) The UCSC genome browser track view of the *CD48* and *CD40* genomic locus and total RNA‐seq data expression of *CD48* and *CD40* mRNA in the cortex of 6‐month WT and 5хFAD. (C) RT‐qPCR analysis of *CD48* and *CD40* mRNA expression in the cortex of 6‐month WT and 5хFAD. The data are presented as mean ± SEM with *n* = 4 mice per group. The *p* values are calculated using unpaired two‐tailed *t*‐test. (D) Western blot analysis showing the CD48 and CD40 protein expression levels in the cortex and hippocampus of 4‐month and 10‐month WT and 5хFAD. Equal protein loading was confirmed by β‐actin in the protein extracts. *p < .05. SEM, standard error of the mean.

To examine the AD‐related gene expression, we performed the total RNA‐seq in the cortex and blood of 6‐month wild‐type (WT) and 5хFAD (Figure 1A). We sorted the 6565 genes in >2‐fold DEGs between WT and 5хFAD as the AD model mouse. Previous studies have been reported that the abnormally expressed several *CD* genes were closely related to AD progression.^24^, ^26^, ^27^ To confirm the expression of *CD*‐related genes, we separated the *CD*‐related genes in the 6565 genes and of them, we confirmed the >2‐fold upregulated 17 genes (Table S2). Due to total RNA‐seq and analysis of the genomic locus of 17 *CD* candidates, *CD48* and *CD40* were shown AD‐specific upregulation in cortex, especially *CD48* was shown the highest rate of increase about 18‐fold compared to other candidates. Moreover, *CD48* has not identified the relation to AD. Although *CD40* has been suggested to have several roles in AD pathology, there is not enough supporting evidence of AD biomarkers such as tau. Besides, CD48 has been reported to enhance CD40‐mediated activation signal in B cells, which revealed the association between CD48 and CD40.^32^ Therefore, we focused on these two genes, as *CD48* and *CD40*, to investigate the function of these genes in AD. We performed GWAS using total RNA‐seq dataset to measure the *CD48* and *CD40* genes expression in the cortex of WT and 5хFAD. As the result, the UCSC genome browser showed that expression levels of *CD48* and *CD40* genes were highly increased by more than about 1730% and 552%, respectively, in the cortex of 5хFAD when compared to the WT, as well as we indicated FPKM values of *CD48* and *CD40* genes (Figure 1B and Table S3). We further confirmed whether the *CD48* and *CD40* mRNA expressions were increased in the brain using the RT‐qPCR in four different 6‐month WT and 5хFAD. The mRNA expression levels of *CD48* and *CD40* were also significantly increased in the cortex of 5хFAD than in WT (Figure 1C). Moreover, the *CD48* mRNA expression level was more dramatically upregulated than the *CD40* mRNA expression level, and the results showed the same tendency as RNA‐seq. To identify whether the *CD48* and *CD40* translational levels were changed by the transcription levels, western blot analysis was performed in the brain of 4‐ and 10‐month WT and 5хFAD. Interestingly, CD48 and CD40 proteins were remarkably increased in the cortex of the 4‐ and 10‐month 5хFAD than in WT, and CDs were elevated in the hippocampus (Figure 1D; the complete western blots are shown in Figure S2). These results indicated that increased *CD48* and *CD40* gene expression led to the upregulation of the translational level in the cortex and hippocampus of the 5хFAD, as well as these protein levels increased in 4‐ and 10‐month.

### 3.2 *CD48* and *CD40* gene expression profiling in AD patient's brain

**FIGURE 2:**
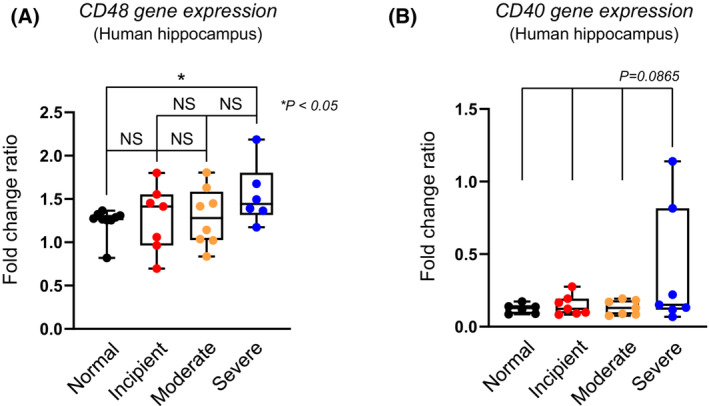
In silico analysis for *CD48* and *CD40* gene expression in AD patients. (A) Analysis of *CD48* gene expression in the human hippocampus microarray dataset of AD patients. Normal group, *n* = 9; incipient group, *n* = 7; moderate group, *n* = 8; and severe group, *n* = 6. (B) Analysis of *CD40* gene expression in the human hippocampus microarray dataset of AD patients. Normal group, *n* = 6; incipient group, *n* = 7; moderate group, *n* = 7; and severe group, *n* = 7. The *p* values are calculated using the post hoc test after one‐way ANOVA. *p < .05. NS, not significant.

Following the discovery of the upregulation of *CD48* and *CD40* gene expression in the AD mouse model, we next performed gene expression profiling for *CD48* and *CD40* in human AD patients' brains to confirm whether the gene expression pattern overlapped between humans and mice. The in silico meta‐analysis was conducted using a human hippocampal tissue‐based microarray data set (GSE1297), with varying severity stages (incipient, moderate and severe) of AD patients aged 75–101 years.^33^ Identification of the fold change ratio of *CD48* and *CD40* gene expression in the human hippocampus revealed that each gene elevated gene expression in severe AD patients group compared to normal group. Interestingly, the *CD48* gene was significantly more expressed in patients in the severe stage of AD patients than in normal group (Figure 2A), and the *CD40* gene showed an upregulated trend, but this was not significant (Figure 2B). However, there were no significant differences between the AD stages (incipient, moderate, and severe) in both *CD48* and *CD40* gene expression. These data correlate the results from the AD mouse model to human patients' data set and demonstrate that both *CD48* and *CD40* genes are substantially upregulated in the severe stage of AD.

### 3.3 Identification and development of AD diagnosis with *CD48* and *CD40*

We found that *CD48* and *CD40* gene expression levels were significantly increased about 441% and 576%, respectively in AD mouse model blood through total RNA‐seq, and showed FPKM values of *CD48* and *CD40* genes (Figure S1A and Table S3). Although several methods have been used for the diagnosis of AD, it has been difficult to make the rapid diagnosis with biopsy through the sample of brain tissue from AD patients.^28^, ^34^ In this study, we developed a novel AD diagnosis method that can rapidly detect *CD48* and *CD40* gene expression levels using multiplex PCR. We further performed our developed multiplex RT‐PCR to evaluate the mRNA expression level of *CD48* and *CD40* in the blood of three different the WT and 5хFAD. Interestingly, we detected *CD48* and *CD40* mRNA expression levels in AD mouse model blood using a multiplex RT‐PCR experiment with specifically designed primers and PCR conditions (Figure S1B,C). These results indicate the potential of multiplex RT‐PCR targeting *CD48* and *CD40* genes as novel diagnostic methods.

### 3.4 *CD48* and *CD40* boost intercellular tau aggregation

**FIGURE 3:**
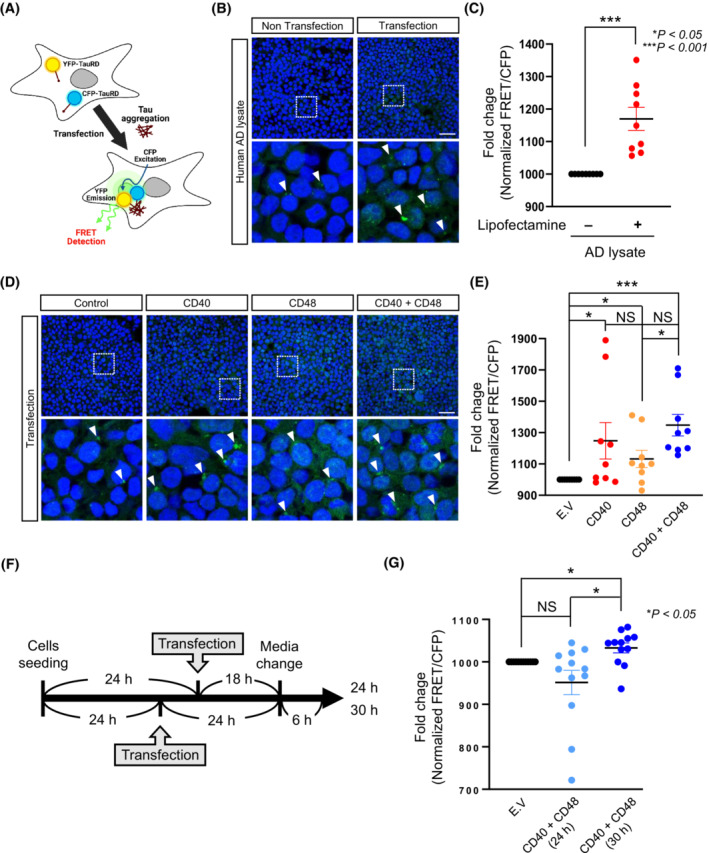
FRET signal from *CD48* and *CD40*‐related intercellular tau aggregation. (A) Schematic representation of the FRET analysis using tau biosensor cells. Aggregation of tau can be detected by the presence of FRET signal. (B) Representative images of tau biosensor cells transfected into the brain lysate of AD patients with or without lipofectamine. Fluorescent microscope images of seeded biosensor cells taken in the GFP channel at 400× magnification. Scale bar = 50 μm. (C) FRET signal measured by a microplate reader. (D) Representative images of tau biosensor cells transfected with each construct of *CD48* or *CD40* with lipofectamine. Fluorescent microscope images of seeded biosensor cells taken in the GFP channel at 400× magnification. Scale bar = 50 μm. (E) FRET signal measured by a microplate reader. (F) Schematic representation of the experimental timeline. The tau biosensor cells were transfected with *CD48* and *CD40* in a time‐dependent manner (24 h and 30 h). (G) FRET signal measured by a microplate reader. CFP signal was used to normalize the FRET signal. The data are presented as mean ± SEM, with (C and E) *n* = 9 and (G) *n* = 12 biological replicates. The *p* values are calculated using unpaired two‐tailed *t*‐test. *p < .05, **p < .01, and ***p < .001.

Previous studies have reported that several *CD* genes are closely related to AD progression, including the accumulation of Aβ and neuro‐inflammation.^26^, ^27^ However, the relationship between tau aggregation and *CDs* has not yet been investigated. To evaluate whether tau aggregation was associated with *CD48* and *CD40*, we performed the FRET analysis using tau biosensor cells (Figure 3A).^29^, ^35^, ^36^ As a positive control, we treated the human brain lysate from AD patients in tau biosensor cells to ensure tau aggregation from FRET activity.^37^ The lipofectamine‐treated AD lysate group showed significantly increased tau aggregation, as visualized by FRET activity (Figure 3B). We consistently measured the FRET signal, which resulted in remarkably higher levels than the control (Figure 3C). Based on these results, we transfected *CD48* and *CD40* into the tau biosensor cells. Interestingly, the *CD48* and *CD40* co‐transfected groups showed more intracellular FRET‐positive tau aggregates than the other groups, and each *CD48* or *CD40* transfected group exhibited increased aggregation of tau compared to the control (Figure 3D). Furthermore, the FRET signal was significantly increased in the overexpressed *CD48* and *CD40* groups when compared to other groups, and single overexpressed *CD48* or *CD40* groups also showed an elevated FRET signal compared to the control (Figure 3E). Although the overexpressed *CD40* group showed a higher FRET signal than the overexpressed *CD48* group, there was no significant difference (*p* = .3817). Taken together, both *CD48* and *CD40* contributed to intracellular tau aggregation and had a greater boosting effect in tau aggregation than either *CD48* or *CD40* alone, that show the relation to co‐overexpressed *CD48* and *CD40* in AD patients and mice.

We also hypothesized that upregulated *CD48* and *CD40* affected tau aggregation in a time‐dependent manner. To test this hypothesis, we designed an experimental timeline (Figure 3F). We found that *CD48* and *CD40* overexpression in 30 h significantly elevated the FRET signal compared to other groups (Figure 3G). However, *CDs* overexpression in 24 h was not significantly different from that of the control. These results demonstrated that increased *CD48* and *CD40* promoted tau aggregation in a time‐dependent manner. Therefore, both *CD48* and *CD40* contributed to AD progression through tau aggregation.

### 3.5 *CD48* and *CD40* activate the NF‐κB signaling pathway

**FIGURE 4:**
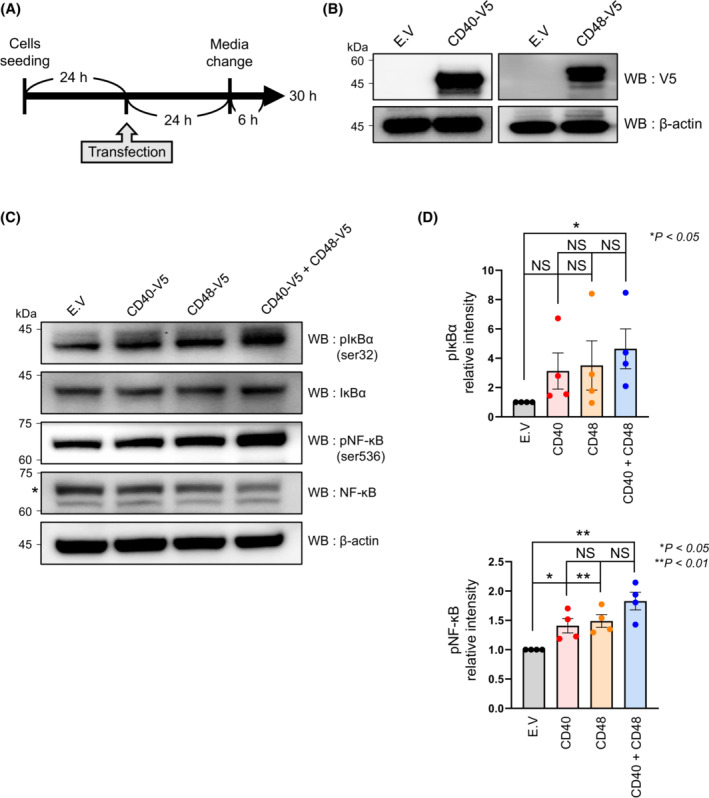
Activation of NF‐ĸB signaling pathway by *CD48* and *CD40* overexpression. (A) Schematic representation of the experimental timeline. The tau biosensor cells were transfected with *CD48* and *CD40* and incubated total 30 h. The cells were transfected with V5‐tagged *CD48* and *CD40* to examine the protein expression levels using western blot by detecting V5 (B), pIĸBα, IĸBα, pNF‐ĸB, and NF‐ĸB antibodies (C). (D) Expression intensity of NF‐ĸB signaling pathway examined by ImageJ 1.50i software. Equal protein loading was confirmed by β‐Actin in the protein extracts and normalized to β‐Actin. The data are presented as mean ± SEM, with *n* = 4 biological replicates. The *p* values were calculated using unpaired two‐tailed *t*‐test. Asterisk (*) indicates main signal band. *p < .05, and **p < .01.

NF‐κB signaling pathways are related to various neurodegenerative diseases, especially AD.^38^, ^39^, ^40^ However, there is not enough the evidence of the NF‐κB signaling pathway that relates *CD48* and *CD40* with tau aggregation. Therefore, to examine whether NF‐κB signaling pathways were altered by the overexpression of *CD48* and *CD40*, we assessed the protein expression of NF‐κB signaling pathway using western blot analysis. We transfected *CD48* and *CD40* into the tau biosensor cells (Figure 4A). To demonstrate the transfection, we confirmed the protein expression levels of CD48 and CD40 (Figure 4B; the complete western blots are shown in Figure S3A). Based on these results, we examined the activation of NF‐κB signaling pathways, such as the inhibitor of kappa B alpha (IĸBα) and NF‐κB. Remarkably, IĸBα and NF‐κB were significantly activated as the phosphorylation by both overexpressed *CD48* and *CD40* when compared to the control (Figure 4C,D; the complete western blots are shown in Figure S3B). In addition, we performed the knockdown of *CD48* and *CD40* to examine whether the alteration of NF‐κB signaling pathways. Our used tau biosensor cells are based on HEK293T cells and are not express the endogenous *CD48*. Therefore, we overexpressed *CD48* and *CD40*, before knocking down *CD48* and *CD40*. As the result, pIĸBα was decreased and pNF‐κB was increased compared to scrambled control and co‐overexpressed *CD48* and *CD40* group (Figure S4; the complete western blots are shown in Figure S5). Although the phosphorylated levels were not meaningful differed between the single and both overexpressed *CDs*, both overexpressed *CD48* and *CD40* were show more increased tendency than single overexpressed *CD*. These results indicated that *CD48* and *CD40* were more affected by equal upregulation in AD pathology. Taken together, *CD48* and *CD40* play important roles in AD progression through tau aggregation, which activates the NF‐κB signaling pathway.

## 4 DISCUSSION

Upregulation of *CD40* in AD has been suggested through the analysis of patients' tissues and serum.^41^, ^42^ However, it is unclear whether the effectiveness of *CD40* expression in AD progression is due to the phenotype of genetic interactions of *CD40*. Furthermore, a systematic analysis of the synergistic effect of *CD40* and other *CD* family expression has not yet been reported. We reasoned that unexplored *CDs*, as well as *CD40*, for AD progression may exist and might be valuable targets for biomarkers. Interestingly, the potency of *CD48* and *CD40* co‐expression has not been previously reported. We identified and demonstrated new factors for AD progression through GWAS and showed that there are several *CD* genes, including *CD48 and CD40*, and genetic pathways whose expression boosts tau pathology by activating NF‐κB signaling.

**FIGURE 5:**
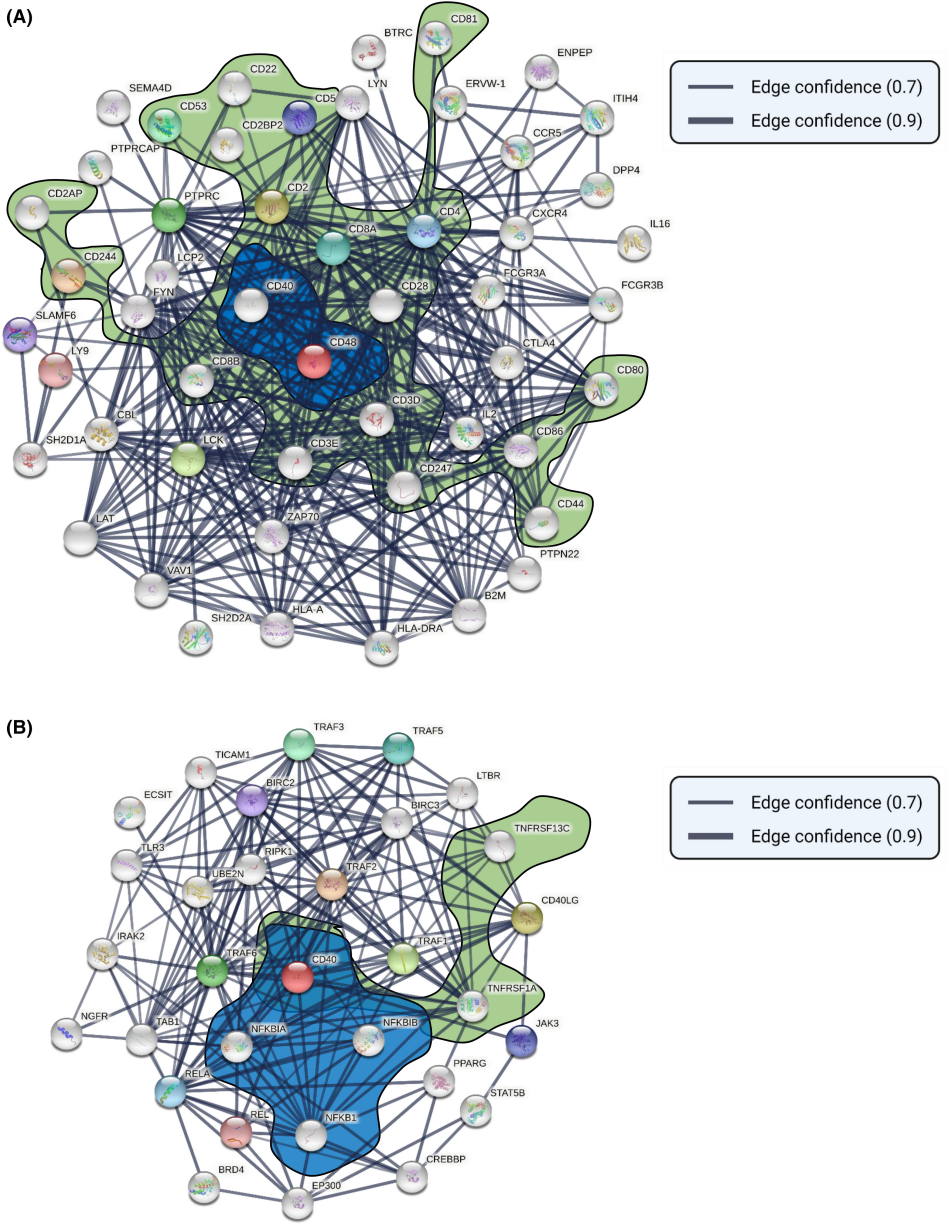
CD48‐ and CD40‐related protein–protein networks. Network interaction diagrams showing the CD48 and CD40 protein‐related proteins in humans. The interaction maps are obtained from the STRING (https://string‐db.org/) database. (A) The CD48 protein‐related protein network. (B) The CD40 protein‐related protein network. The line thickness indicates the strength of data support with confidence cutoff of 0.7 and 0.9.

Mapping of biological interaction from large‐scale proteomic information is often applied to the prediction of protein–protein interaction (PPI) research.^43^ Because this project might be preferable to have access to find specific PPIs in targeted diseases, one or both of the proteins identified in the targeted disease probably demonstrate the possibility of the association of potent intercellular signaling pathways. In addition, PPI mapping revealed direct interactions between the disease‐specific factors on partner proteins. It has previously been suggested that CDs are receptor proteins involved in accelerating Aβ accumulation because the response of CDs leads to Aβ binding in AD progression through signaling transduction and inflammatory responses.^44^ Despite the fact that *CDs* are assumed to be risk factors for AD due to their association with Aβ binding, we found that tau aggregation is boosted by the upregulation of *CDs*. Further, we could not show the intermediation of CDs and well‐known AD‐associated receptors such as toll‐like receptor or G‐protein coupled receptor to distinguish between Aβ and tau pathology. However, overexpression of *CDs* suggests that there is a distinct feature of *CDs* that is responsible for the activation of NF‐κB signaling, which is strongly involved in tau aggregation (Figure 4). In addition, mapping of CD48 and CD40 protein networking showed that diverse *CDs* were associated with tumor necrosis factor receptors (Figure 5). This analysis suggests that the upregulation of *CDs* may activate and accelerate the NF‐κB signaling pathway, implying that *CD48* and *CD40* might exacerbate tau aggregation. On the other hand, the knockdown of *CD48* and *CD40* showed the decrease of pIĸBα expression level compared to scrambled control and co‐overexpressed *CD48* and *CD40* group. Unexpectedly, pNF‐κB was expressed higher than scrambled control and co‐overexpressed *CD48* and *CD40* group. These results suggested that the *CD48* and *CD40* might have the other associated factor for the regulation of NF‐κB, and also have independent work for indicated NF‐κB cascade. However, regarding the intricate cellular mechanism, there remain to be further studies to demonstrate the relations between NF‐κB signaling pathways and downregulated *CD48* and *CD40*.

**FIGURE 6:**
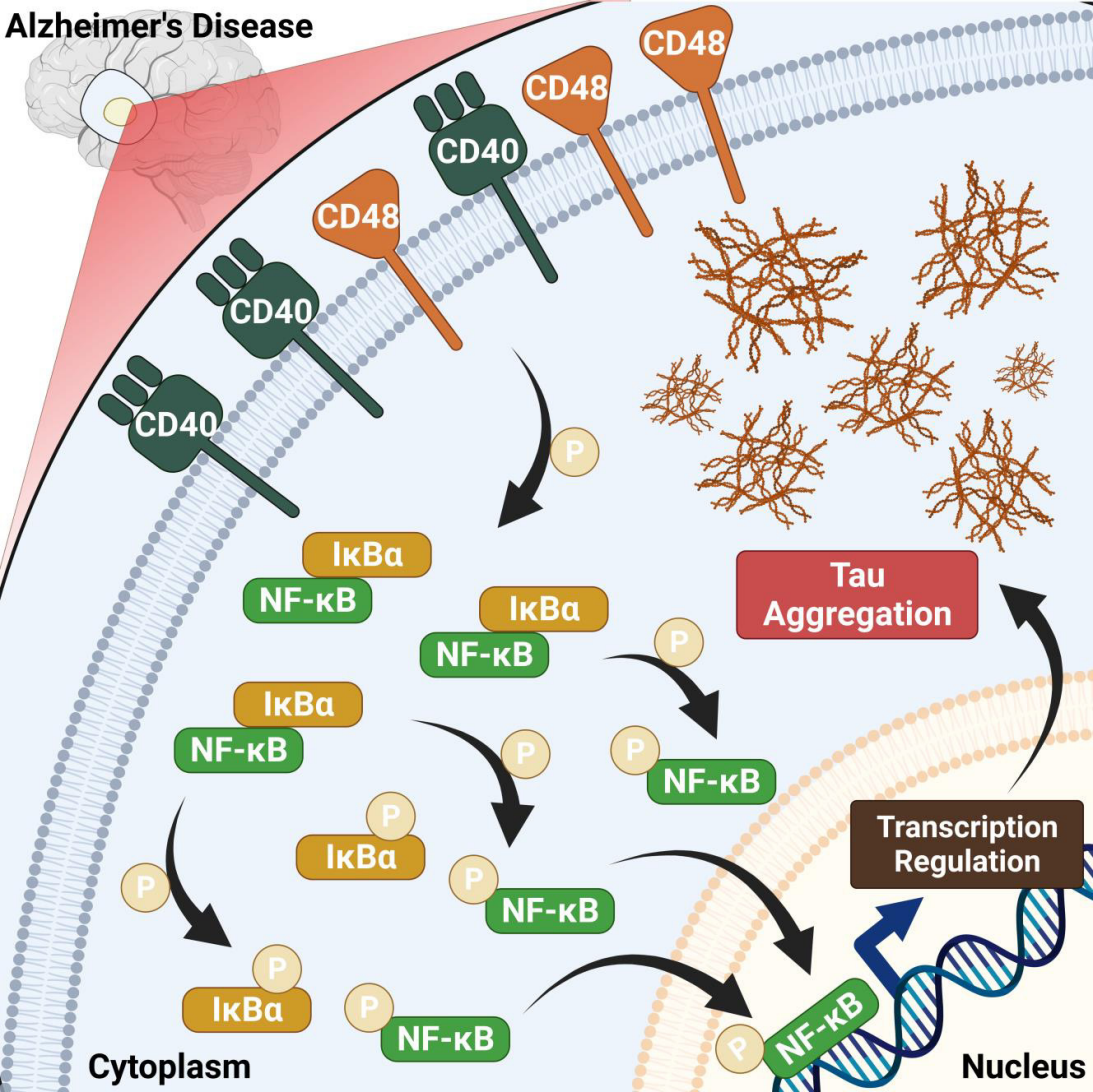
Mechanism of *CD48* and *CD40* in AD. The illustration shows the summary of the crucial roles of *CD48* and *CD40* in AD progression. In AD, upregulated *CD48* and *CD40* promoted tau aggregation though the activation of IĸBα, that is, phosphorylation that phosphorylated IĸBα was separated from NF‐ĸB, and the liberated NF‐ĸB translocated to the nucleus and promoted the regulation of transcription.

**FIGURE 7:**
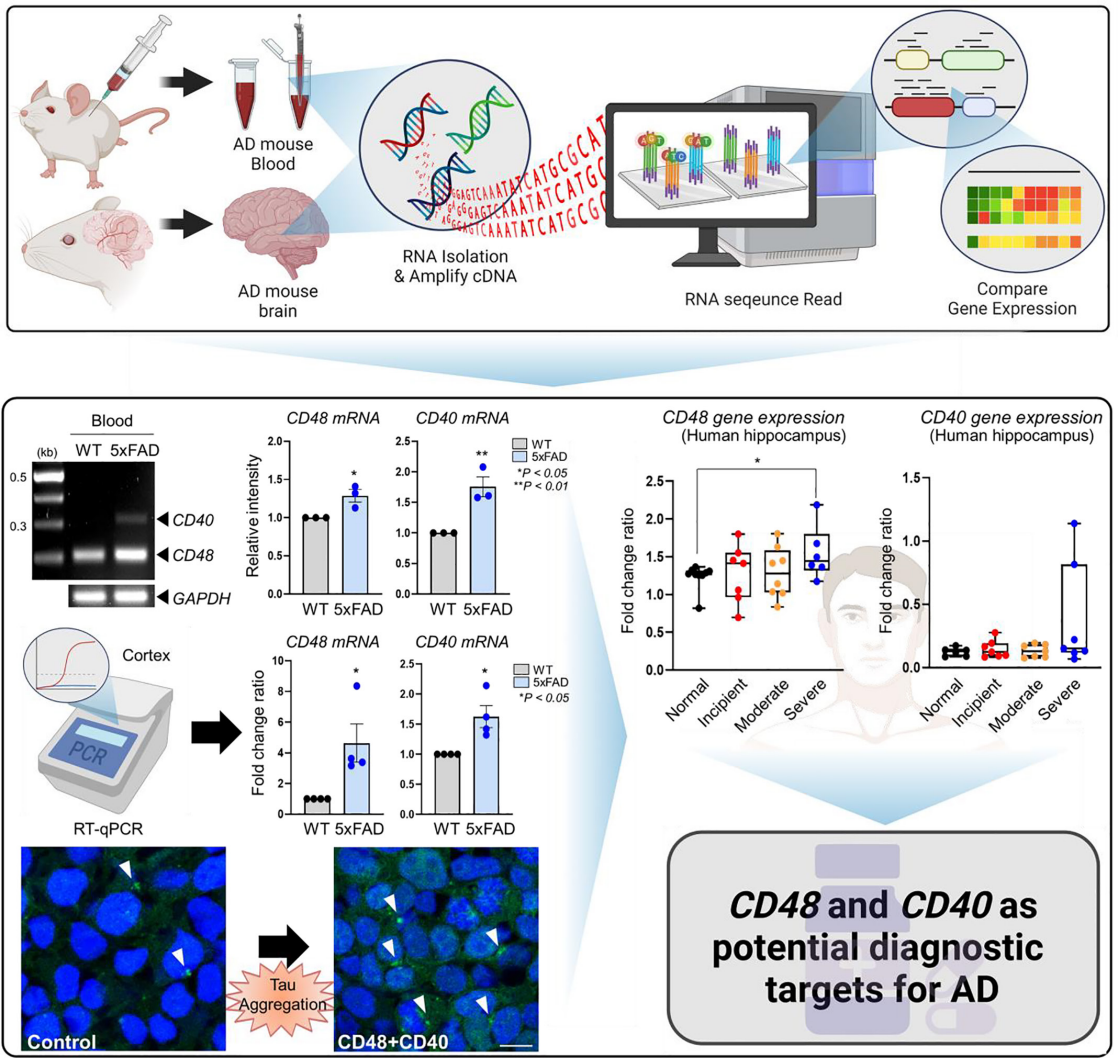
Graphical abstract. These graphical diagram show the main finding of our present study. The *CD48* and *CD40* genes are upregulated in Alzheimer's disease (AD) pathogenesis, which associated with boosting the tau aggregation.

Focusing on *CDs* as new genetic factors of AD, we showed that *CD48* and *CD40* are strongly associated with the phenotype of tau pathology. Indeed, several genetic factors in AD have gain‐of‐function, resulting in clinical symptoms.^13^ We validated *CD48* and *CD40* as targets of AD diagnosis and showed using a novel cell‐based FRET method that expression of *CDs* boosted tau aggregation in a time‐dependent manner through the activation of NF‐κB signaling (Figure 6). It is important to recognize that inhibition or depletion of *CD48* and *CD40* in the AD model may have a potential role in disturbing tau aggregation. Many of the liquid biopsies in current clinical use have been developed to target AD, and an understanding of genetic factors and potential protein interactions are important for diagnosis. In both AD models and patients, we observed the genetic aspects of *CD* expression patterns during AD progression. This result indicated the possibility that *CD48* and *CD40* can be diagnostic markers in AD model (Figure 7), further investigation with AD‐patient is required in order to convince clinical diagnostics and wide range of biomarkers such as disease developmental stage dependent markers.

Previous studies have suggested that failure of neuroinflammatory response against dementia leads to the aggregation of Aβ fibrils.^45^, ^46^, ^47^ This Aβ pathogenesis cause tau‐associated lesions such as tau tangle accumulations, which show neurotoxic and disease progression.^46^ Based on these contexts, our results may have the potential, by which mechanism initiates through Aβ accumulation. However, there are two possibilities has been suggested that Aβ can be upstream of tau aggregation in AD and toxic tau also effected to Aβ toxicity via a feedback loop.^48^ Additionally, according to the subjects in AD study (GSE1297), the neurofibrillary tangle count was shown to be elevated along the severity; Control (27 ± 1.0), Incipient (94 ± 1.8), Moderate (256 ± 3.5) and Severe (327 ± 7.2).^49^ Thus, *CD48* and *CD40* gene expression in severe human hippocampus (Figure 2), there possibly be reflected subjects' characters. In addition, CD48 and CD40 also increased the protein expression in 4‐month 5xFAD (Figure 1D). Those counts also carefully deduce that increased *CD48* and *CD40* gene expression in the severe phase was not only the consequence of the later stage. Taken together, it has potential interest that the identification of the important roles of *CD48* and *CD40* in tau aggregation. Further studies based on experiments with the new AD‐animal model and individual AD patient analysis might be explained their important role in AD pathology. Moreover, recent advances in deep‐learning analysis have improved the prediction of genetic alterations in target diseases.^50^, ^51^ Further studies will be needed to characterize the genetic mutation of *CDs* in individual AD patients. The identification of other cryptic mutations in *CD* genes from patients with AD may elucidate the molecular mechanisms of AD progression.

## 5 CONCLUSION

In conclusion, the present study elucidates the interaction between the upregulation of transmembrane genes and AD pathology. Through the gene expression profiles, we present the evidence that *CD* genes of transmembrane protein might be potent biomarker for AD. Furthermore, our results demonstrate the remarkable mechanism in AD progression that overexpressed *CD* genes are closely connected the increase of tau aggregation through the NF‐κB signaling pathway, as well as *CD48* is boosting the aggregation of tau when the *CD40* exerts together. In additions, our developed multiplex RT‐PCR using the *CD genes*, was designed and optimized to diagnose rapidly and simultaneously, which can support the clinical application in diagnosis of AD. However, there are some remaining questions to be investigated. For example, what are the transcription regulation factors related to *CD* genes via NF‐κB signaling pathway. Indeed, further studies will have to identify the correlation of other transmembrane factors or genetic mutations in *CDs*, which may provide more confident molecular mechanisms for AD. Although *CD* genes need to be further studied, *CD48* and *CD40* as the remarkable AD‐related transmembrane protein genes have a potential to be one of the valuable biomarkers or therapeutic factors for the AD.

## AUTHOR CONTRIBUTIONS

Sung‐Hyun Kim, Key‐Hwan Lim, Sumin Yang and Jae‐Yeol Joo performed the experiments, analyzed the data, prepared the figures and commented on the manuscript; Jae‐Yeol Joo and Key‐Hwan Lim designed the research; Jae‐Yeol Joo supervised the whole project; All authors have read and approved the published version of the manuscript.

## DISCLOSURES

The authors declare that they have no competing of interests.

## Supporting information


Figure S1

Figure S2

Figure S3

Figure S4

Figure S5

Table S1

Table S2

Table S3

## Data Availability

Main data are included in this manuscript. The data will be made available on request.
