# Supplementary material for: Boosting of tau protein aggregation by CD40 and CD48 gene expression in Alzheimer's disease
Source: FASEB J. 2022 Dec 15;37(1):e22702. doi: 10.1096/fj.202201197R (PMC13281844; doi:10.1096/fj.202201197R)
Supplement: Supplementary file 1 — Figure S1 [file FSB2-37-e22702-s003.pptx]

## Slide 1
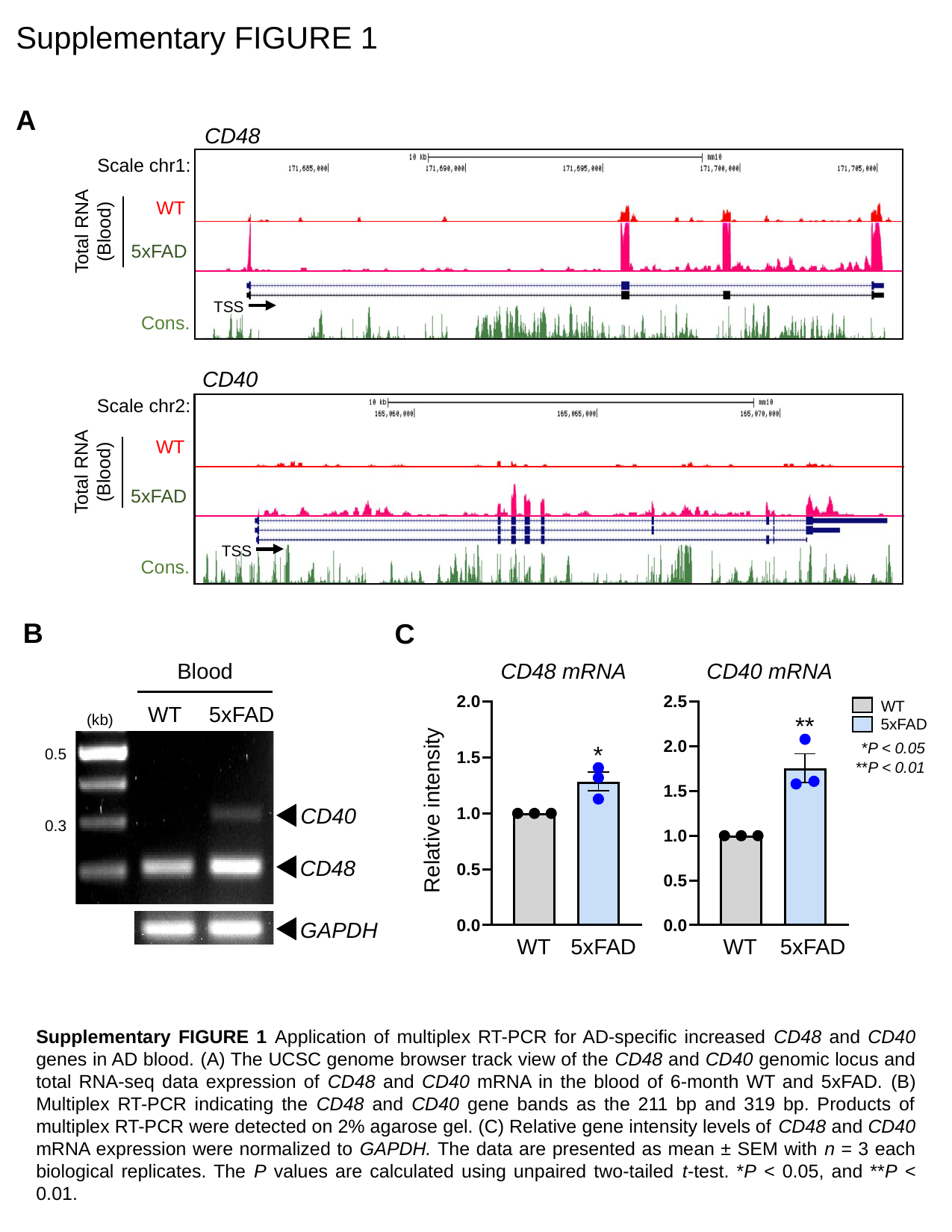

Supplementary FIGURE 1
A
CD48
Scale chr1:
WT
Total RNA
(Blood)
5xFAD
TSS
Cons.
CD40
Scale chr2:
WT
Total RNA
(Blood)
5xFAD
TSS
Cons.
B
C
Blood
CD48 mRNA
CD40 mRNA
WT
WT
5xFAD
(kb)
**
5xFAD
*P < 0.05
*
0.5
**P < 0.01
Relative intensity
CD40
0.3
CD48
GAPDH
WT
5xFAD
WT
5xFAD
Supplementary FIGURE 1 Application of multiplex RT-PCR for AD-specific increased CD48 and CD40 genes in AD blood. (A) The UCSC genome browser track view of the CD48 and CD40 genomic locus and total RNA-seq data expression of CD48 and CD40 mRNA in the blood of 6-month WT and 5хFAD. (B) Multiplex RT-PCR indicating the CD48 and CD40 gene bands as the 211 bp and 319 bp. Products of multiplex RT-PCR were detected on 2% agarose gel. (C) Relative gene intensity levels of CD48 and CD40 mRNA expression were normalized to GAPDH. The data are presented as mean ± SEM with n = 3 each biological replicates. The P values are calculated using unpaired two-tailed t-test. *P < 0.05, and **P < 0.01.
