# Supplementary material for: Boosting of tau protein aggregation by CD40 and CD48 gene expression in Alzheimer's disease
Source: FASEB J. 2022 Dec 15;37(1):e22702. doi: 10.1096/fj.202201197R (PMC13281844; doi:10.1096/fj.202201197R)
Supplement: Supplementary file 2 — Figure S2 [file FSB2-37-e22702-s001.pptx]

## Slide 1
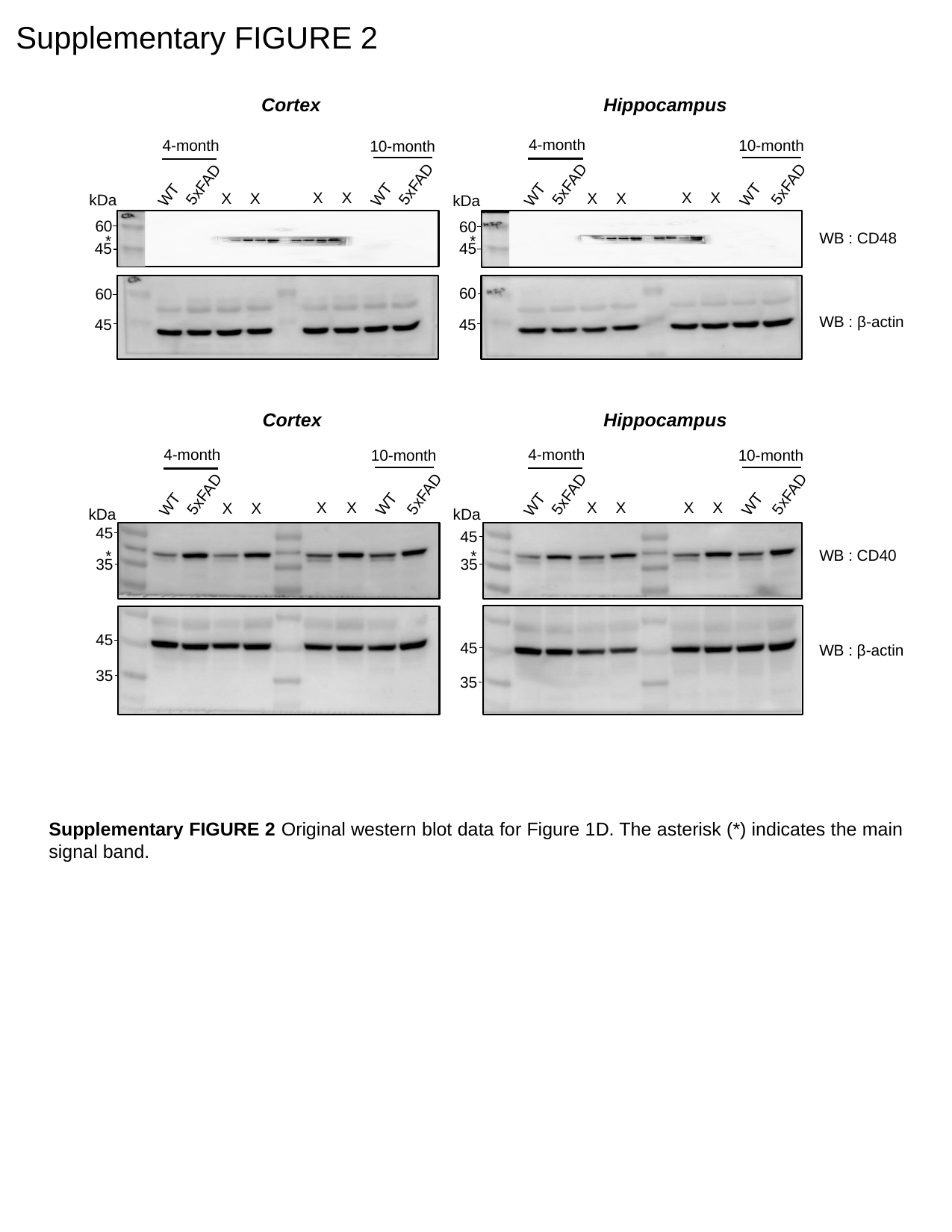

Supplementary FIGURE 2
Hippocampus
Cortex
4-month
4-month
10-month
10-month
5хFAD
5хFAD
5хFAD
5хFAD
WT
WT
WT
WT
X
X
X
X
X
X
X
X
kDa
kDa
60
60
WB : CD48
 *
 *
45
45
60
60
WB : β-actin
45
45
Hippocampus
Cortex
4-month
4-month
10-month
10-month
5хFAD
5хFAD
5хFAD
5хFAD
WT
WT
WT
WT
X
X
X
X
X
X
X
X
kDa
kDa
45
45
WB : CD40
 *
 *
35
35
45
45
WB : β-actin
35
35
Supplementary FIGURE 2 Original western blot data for Figure 1D. The asterisk (*) indicates the main signal band.
