# Supplementary material for: Boosting of tau protein aggregation by CD40 and CD48 gene expression in Alzheimer's disease
Source: FASEB J. 2022 Dec 15;37(1):e22702. doi: 10.1096/fj.202201197R (PMC13281844; doi:10.1096/fj.202201197R)
Supplement: Supplementary file 3 — Figure S3 [file FSB2-37-e22702-s007.pptx]

## Slide 1
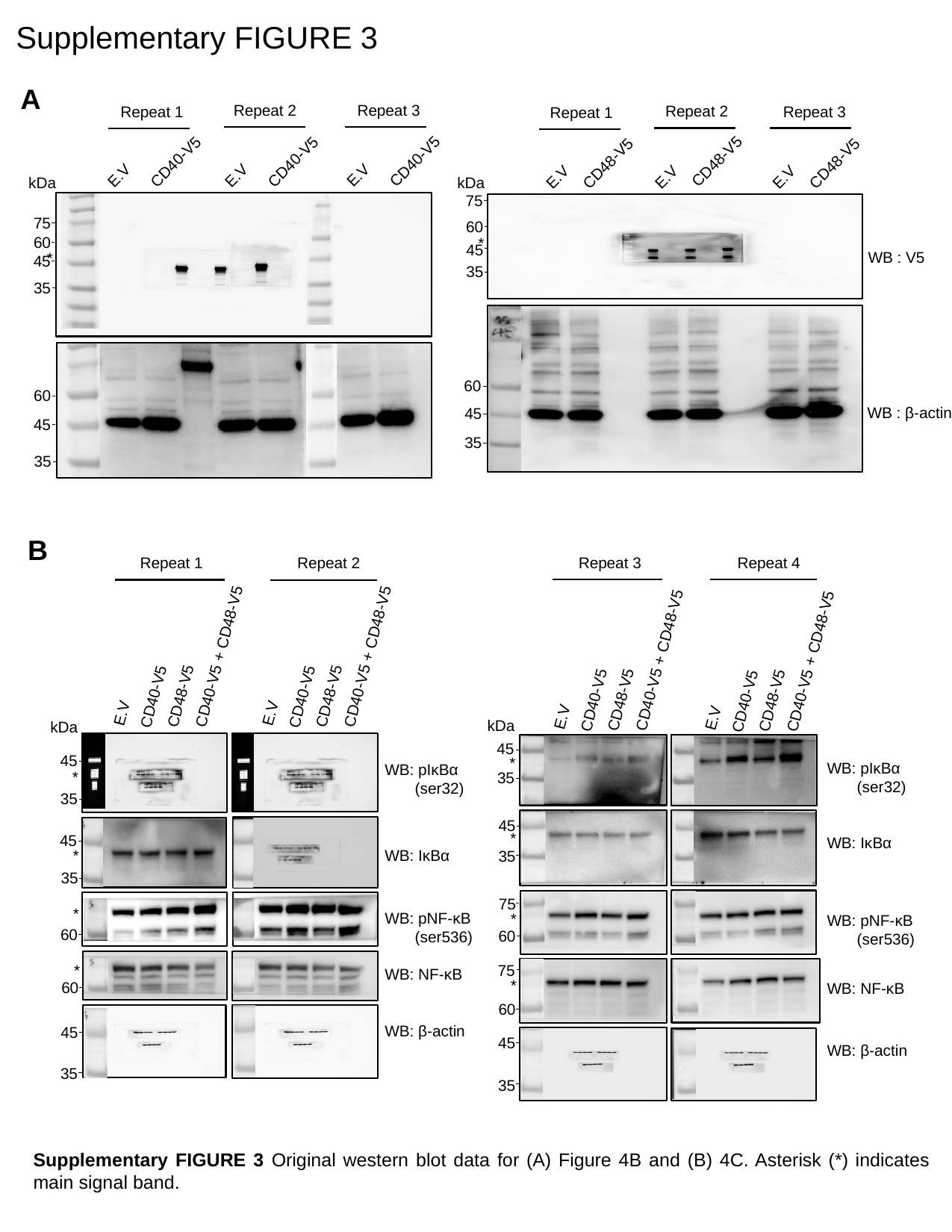

Supplementary FIGURE 3
A
Repeat 2
Repeat 3
Repeat 2
Repeat 3
Repeat 1
Repeat 1
CD40-V5
CD40-V5
CD40-V5
CD48-V5
CD48-V5
CD48-V5
E.V
E.V
E.V
E.V
E.V
E.V
kDa
kDa
75
75
60
60
 *
45
WB : V5
 *
45
35
35
60
60
WB : β-actin
45
45
35
35
B
Repeat 3
Repeat 4
Repeat 1
Repeat 2
CD40-V5 + CD48-V5
CD40-V5 + CD48-V5
CD40-V5 + CD48-V5
CD40-V5 + CD48-V5
CD48-V5
CD48-V5
CD40-V5
CD40-V5
CD48-V5
CD40-V5
CD48-V5
CD40-V5
E.V
E.V
E.V
E.V
kDa
kDa
 45
 45
 *
WB: pIĸBα
 (ser32)
WB: pIĸBα
 (ser32)
 *
 35
 35
 45
 35
 *
 45
WB: IĸBα
WB: IĸBα
 *
 35
 75
 *
 60
WB: pNF-ĸB
 (ser536)
 *
WB: pNF-ĸB
 (ser536)
 60
 75
 *
WB: NF-ĸB
 *
 60
WB: NF-ĸB
 60
WB: β-actin
 45
 45
WB: β-actin
 35
 35
Supplementary FIGURE 3 Original western blot data for (A) Figure 4B and (B) 4C. Asterisk (*) indicates main signal band.
