# Supplementary material for: Boosting of tau protein aggregation by CD40 and CD48 gene expression in Alzheimer's disease
Source: FASEB J. 2022 Dec 15;37(1):e22702. doi: 10.1096/fj.202201197R (PMC13281844; doi:10.1096/fj.202201197R)
Supplement: Supplementary file 4 — Figure S4 [file FSB2-37-e22702-s005.pptx]

## Slide 1
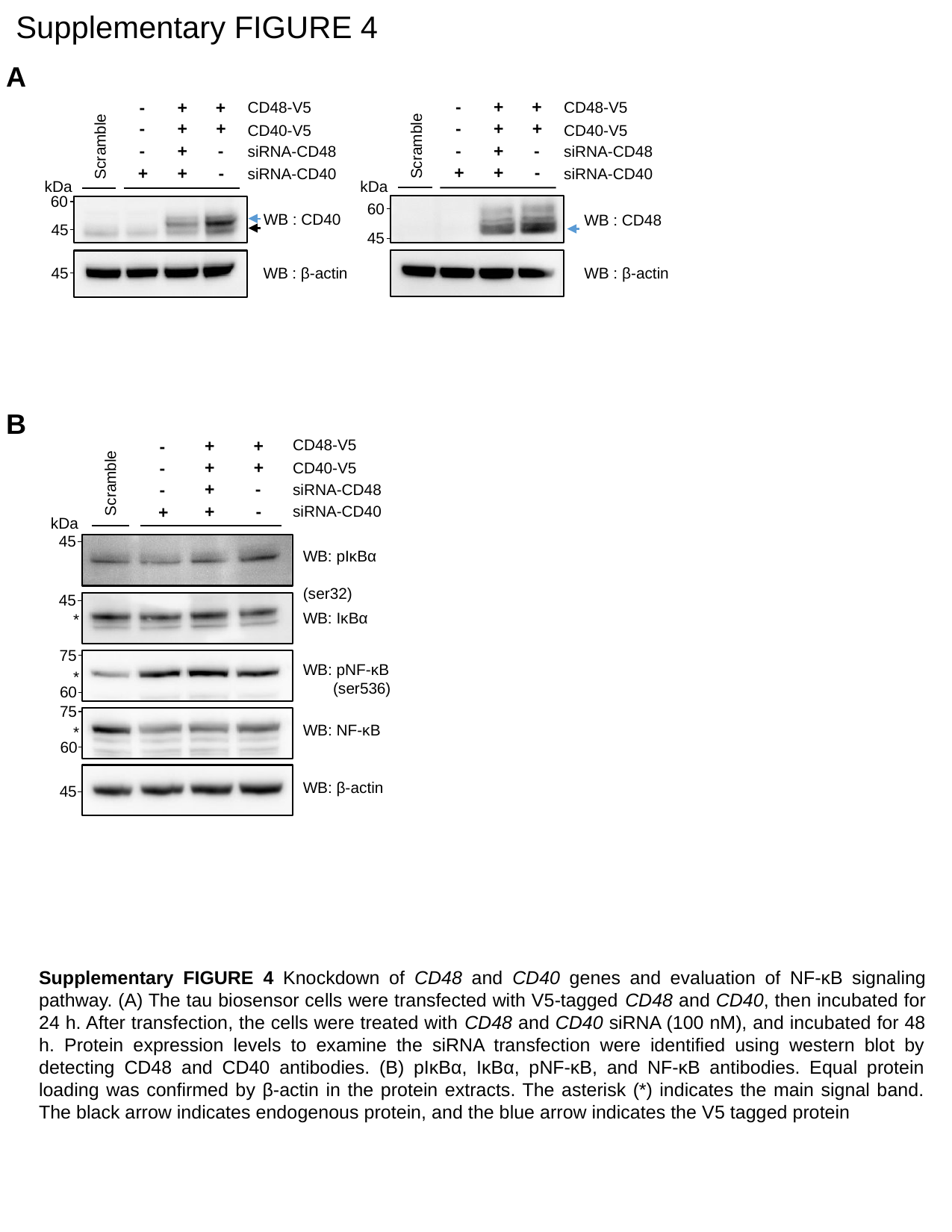

Supplementary FIGURE 4
A
-
+
+
CD48-V5
-
+
+
CD40-V5
Scramble
-
+
-
siRNA-CD48
+
+
-
siRNA-CD40
-
+
+
CD48-V5
-
+
+
CD40-V5
Scramble
-
+
-
siRNA-CD48
+
+
-
siRNA-CD40
kDa
kDa
60
60
WB : CD40
WB : CD48
45
45
WB : β-actin
WB : β-actin
45
B
+
+
CD48-V5
-
+
+
-
CD40-V5
Scramble
+
-
-
siRNA-CD48
+
-
+
siRNA-CD40
kDa
45
WB: pIĸBα
 (ser32)
45
WB: IĸBα
 *
75
WB: pNF-ĸB
 (ser536)
 *
60
75
WB: NF-ĸB
 *
60
WB: β-actin
45
Supplementary FIGURE 4 Knockdown of CD48 and CD40 genes and evaluation of NF-ĸB signaling pathway. (A) The tau biosensor cells were transfected with V5-tagged CD48 and CD40, then incubated for 24 h. After transfection, the cells were treated with CD48 and CD40 siRNA (100 nM), and incubated for 48 h. Protein expression levels to examine the siRNA transfection were identified using western blot by detecting CD48 and CD40 antibodies. (B) pIĸBα, IĸBα, pNF-ĸB, and NF-ĸB antibodies. Equal protein loading was confirmed by β-actin in the protein extracts. The asterisk (*) indicates the main signal band. The black arrow indicates endogenous protein, and the blue arrow indicates the V5 tagged protein
