# Supplementary material for: Boosting of tau protein aggregation by CD40 and CD48 gene expression in Alzheimer's disease
Source: FASEB J. 2022 Dec 15;37(1):e22702. doi: 10.1096/fj.202201197R (PMC13281844; doi:10.1096/fj.202201197R)
Supplement: Supplementary file 5 — Figure S5 [file FSB2-37-e22702-s006.pptx]

## Slide 1
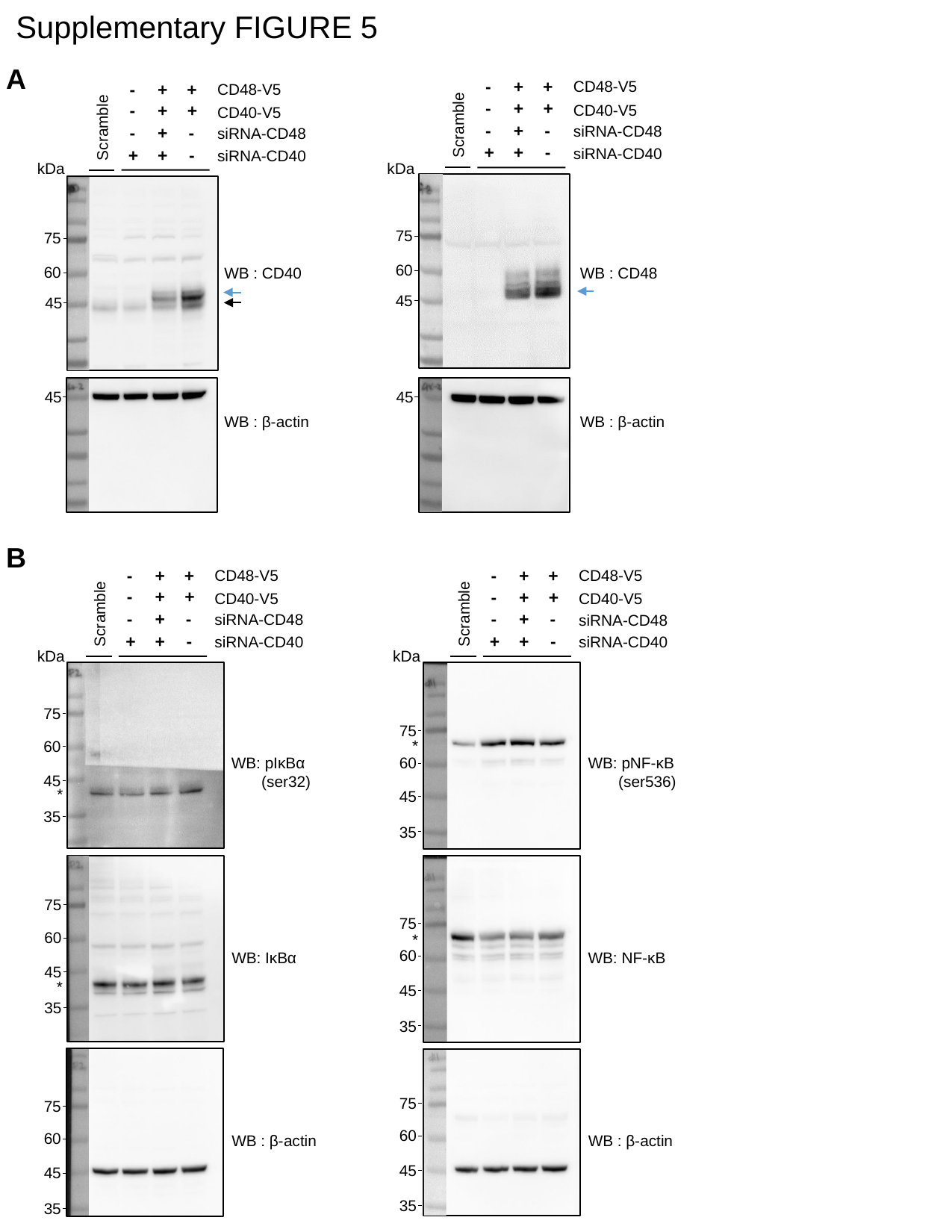

Supplementary FIGURE 5
A
-
+
+
CD48-V5
-
+
+
CD40-V5
Scramble
-
+
-
siRNA-CD48
+
+
-
siRNA-CD40
-
+
+
CD48-V5
-
+
+
CD40-V5
Scramble
-
+
-
siRNA-CD48
+
+
-
siRNA-CD40
kDa
kDa
75
75
60
60
WB : CD40
WB : CD48
45
45
45
45
WB : β-actin
WB : β-actin
B
-
+
+
CD48-V5
-
+
+
CD40-V5
Scramble
-
+
-
siRNA-CD48
+
+
-
siRNA-CD40
-
+
+
CD48-V5
-
+
+
CD40-V5
Scramble
-
+
-
siRNA-CD48
+
+
-
siRNA-CD40
kDa
kDa
75
75
 *
60
WB: pIĸBα
 (ser32)
WB: pNF-ĸB
 (ser536)
60
45
 *
45
35
35
75
75
60
 *
60
WB: IĸBα
WB: NF-ĸB
45
 *
45
35
35
75
75
60
60
WB : β-actin
WB : β-actin
45
45
35
35

## Slide 2
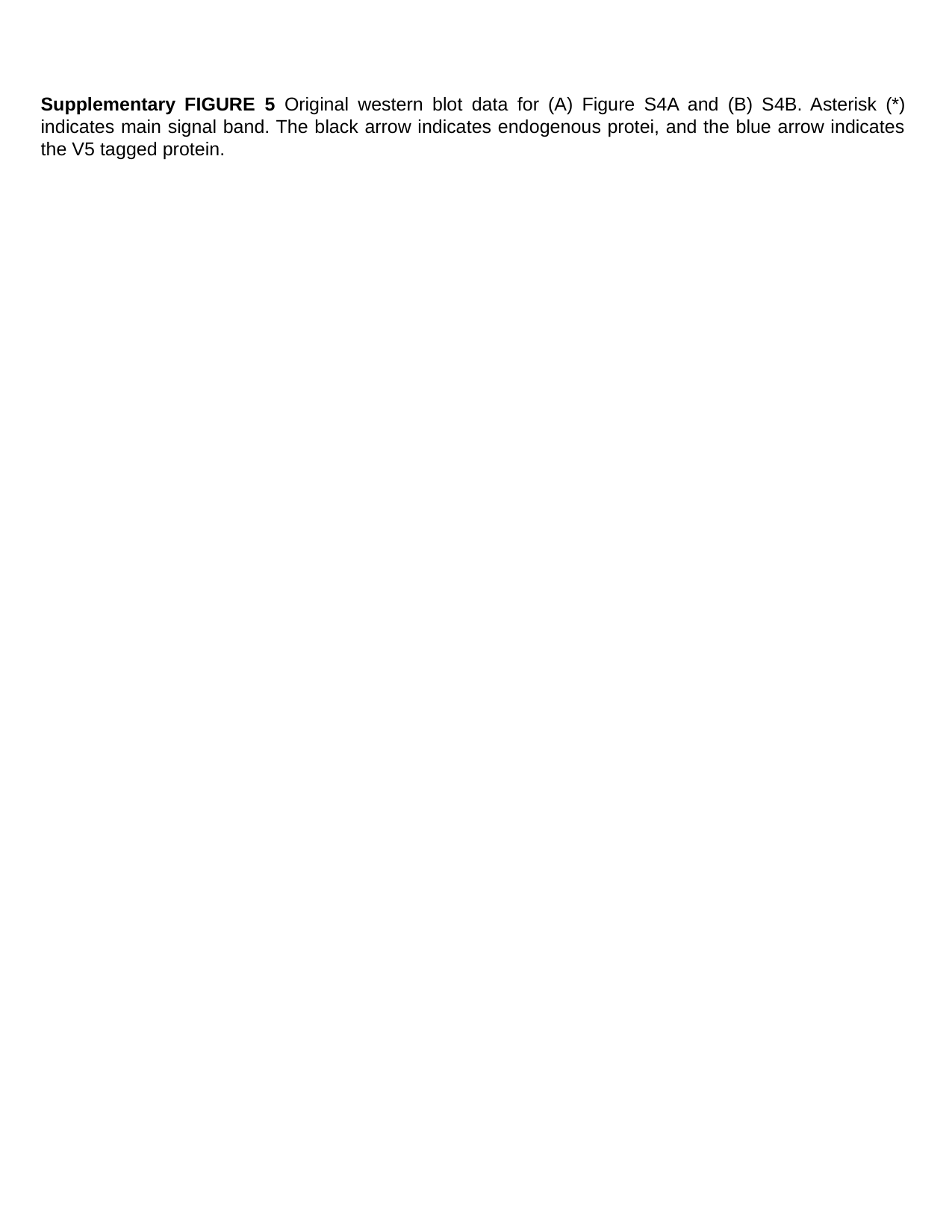

Supplementary FIGURE 5 Original western blot data for (A) Figure S4A and (B) S4B. Asterisk (*) indicates main signal band. The black arrow indicates endogenous protei, and the blue arrow indicates the V5 tagged protein.
