# Supplementary material for: Boosting of tau protein aggregation by CD40 and CD48 gene expression in Alzheimer's disease
Source: FASEB J. 2022 Dec 15;37(1):e22702. doi: 10.1096/fj.202201197R (PMC13281844; doi:10.1096/fj.202201197R)
Supplement: Supplementary file 6 — Table S1 [file FSB2-37-e22702-s008.docx]

**Supplementary Table 1** Subject characteristics in Alzheimer’s disease from GSE1297 expression profiling by array

| **Group** | **Sex** | **Age** | **PMI** | **BRAAK** | **NFT** | **MMSE** |
| --- | --- | --- | --- | --- | --- | --- |
| Control | Male | 75 | 3.35 | 1 | 0.4 | 29 |
|  |  | 77 | 3.5 | 1 | 0.3 | 28 |
|  |  | 80 | 3 | 4 | 0.4 | 26 |
|  |  | 80 | 2.25 | 2 | 7 | 26 |
|  |  | 85 | 2 | 3 | 8 | 30 |
|  |  | 87 | 2 | 2 | 1.3 | 27 |
|  |  | 95 | 1.75 | 1 | 0 | 28 |
|  | Female | 92 | 2.45 | 2 | 2 | 26 |
|  |  | 97 | 2.75 | 3 | 4.8 | 29 |
| Incipient | Male | 88 | 2.75 | 6 | 5.5 | 24 |
|  |  | 88 | 2.75 | 5 | 17.4 | 20 |
|  | Female | 83 | 2.25 | 5 | 6.4 | 26 |
|  |  | 91 | 4 | 3 | 7.2 | 25 |
|  |  | 95 | 6.17 | 6 | 12 | 29 |
|  |  | 97 | 4 | 5 | 8 | 21 |
|  |  | 101 | 1.35 | 5 | 65.8 | 25 |
| Moderate | Male | 81 | 3.5 | 6 | 30.6 | 17 |
|  |  | 82 | 4 | 6 | 26.5 | 14 |
|  | Female | 79 | 3 | 6 | 15.8 | 18 |
|  |  | 82 | 2.75 | 5 | 22 | 18 |
|  |  | 83 | 2.53 | 5 | 42 | 17 |
|  |  | 85 | 2.92 | 6 | 35.2 | 18 |
|  |  | 86 | 4.25 | 6 | 13.8 | 15 |
|  |  | 89 | 2.63 | 5 | 19 | 15 |
| Severe | Male | 85 | 2.5 | 6 | 21 | 4 |
|  |  | 93 | 3.08 | 6 | 62.5 | 11 |
|  | Female | 65 | 3.05 | 6 | 58.2 | 5 |
|  |  | 79 | 3 | 6 | 18.5 | 11 |
|  |  | 79 | 3.67 | 6 | 24.6 | 2 |
|  |  | 93 | 3 | 6 | 21.5 | 7 |
|  |  | 94 | 2.72 | 5 | 22.6 | 2 |

PMI, Postmortem interval; BRAAK, Braak stage; NFT, Neuro feedback training; MMSE, Mini mental status examination
