# Supplementary material for: Boosting of tau protein aggregation by CD40 and CD48 gene expression in Alzheimer's disease
Source: FASEB J. 2022 Dec 15;37(1):e22702. doi: 10.1096/fj.202201197R (PMC13281844; doi:10.1096/fj.202201197R)
Supplement: Supplementary file 8 — Table S3 [file FSB2-37-e22702-s002.docx]

**Supplementary Table 3** FPKM value in RNA-seq data of *CD48* and *CD40* genes from cortex and blood

| **Origin** | **Cortex** | | | | **Blood** | | | |
| --- | --- | --- | --- | --- | --- | --- | --- | --- |
| Gene | *CD48* | | *CD40* | | *CD48* | | *CD40* | |
| Sample | WT | 5xFDA | WT | 5xFAD | WT | 5xFAD | WT | 5xFAD |
| FPKM value | 0.044713 | 0.773495 | 0.01 | 0.055236 | 1.64765 | 7.26066 | 0.236987 | 1.36498 |
| Normalize to WT of FPKM value | - | 1729.90 | - | 552.36 | - | 440.67 | - | 575.97 |
